# Long-term prognosis and risk factors in tricuspid valve replacement surgery: a single-center study

**DOI:** 10.3389/fsurg.2025.1532945

**Published:** 2025-04-08

**Authors:** Yingjie Ke, Linbin Hua, Shanwen Pang, Qiuji Wang, Lishan Zhong, Zhenzhong Wang, Kan Zhou, Rong Zeng, Huanlei Huang

**Affiliations:** ^1^ Department of Cardiovascular Surgery, Guangdong Provincial People’s Hospital (Guangdong Academy of Medical Sciences), Southern Medical University, Guangzhou, Guangdong, China; ^2^ Department of Cardiovascular Surgery, Guangdong Provincial People’s Hospital’s Nanhai Hospital, The Second People’s Hospital of Nanhai District Foshan City, Foshan, Guangdong, China; ^3^ Department of Cardiovascular Surgery, Guangdong Provincial First Veterans Hospital, Guangzhou, China; ^4^ School of Medicine, South China University of Technology, Guangzhou, China

**Keywords:** tricuspid valve replacement, bioprosthetic valve, Mechanical Valve, long-term follow-up, risk factors

## Abstract

**Background:**

Tricuspid valve replacement (TVR), although accounting for a minority of heart valve surgeries, poses significant challenges, including poor patients’ condition, prosthetic complications, and increased perioperative mortality rates. Despite preferences for valvuloplasty, some cases necessitate replacement. The choice of tricuspid valve type remains controversial, and there is no consensus on surgical risk factors. Additionally, long-term follow-up reports on a large number of cases are lacking. In this study, we aimed to analyze the medical records of the largest number of patients who underwent TVR surgery.

**Methods:**

Patients who underwent TVR between 1999 and 2023 were divided into mechanical (MTVR) and bioprosthetic (BTVR) groups. Risk factors for overall mortality were analyzed.

**Results:**

In total, 626 patients were enrolled. The in-hospital and overall mortality rates were 12.1% and 42.8%, respectively. The in-hospital mortality rate (7.0% vs. 14.2%), incidence of acute renal insufficiency (4.3% vs. 12.2%), and hemodialysis rate (3.2% vs. 10.4%) were significantly higher in the BTVR group than in the MTVR group (*P* < 0.01). The median follow-up was 11 years (range 0.1–24 years). The MTVR group had significantly higher rates of long-term survival, hemorrhagic events, heart failure events, and re-operation rates than the BTVR group (*P* < 0.01). Multifactorial logistic regression analysis identified body weight, New York heart function classification, extracorporeal circulation time, and ventilator time as independent risk factors for in-hospital mortality. New York heart function classification during follow-up was identified as an independent risk factor for overall patient mortality.

**Conclusions:**

MTVR was superior to BTVR regarding short- and long-term outcomes. New York heart function classification was associated with short- and long-term mortality.

## Introduction

Tricuspid valve replacement (TVR) surgery constitutes a relatively small proportion of heart valve surgeries; however, it presents numerous challenges, such as poor patients' condition, complications associated with prosthetic valves, and elevated perioperative mortality rates (1, 2). Given these challenges, both domestic and international experts have favored tricuspid valvuloplasty to achieve favorable long-term clinical outcomes. However, TVR is unavoidable in some cases (3–5). Furthermore, there is a scarcity of long-term follow-up reports on a large number of cases.

In this study, we aimed to analyze the medical records of the largest number of patients who underwent TVR surgery at Guangdong Provincial People's Hospital between 1999 and 2023. By assessing the clinical outcomes of mechanical and biological valve replacement during the perioperative and long-term follow-up periods, we investigated the independent risk factors affecting the short- and long-term prognoses of TVR surgery. To our knowledge, this study represented the largest single-center cohort to date comparing MTVR and BTVR outcomes (*n* = 626). This scale minimizes inter-institutional variability in surgical protocols and postoperative care, thereby providing a unique opportunity to isolate the impact of valve type on long-term survival and complications (3, 6).

## Patients and methods

### Patients

The inclusion criteria were patients undergoing either first-time or repeat TVR during the same period, regardless of concurrent cardiac surgery; for patients who underwent repeat TVR, data from the most recent operations were used.

The exclusion criteria included patients with severe tricuspid regurgitation secondary to primary pulmonary hypertension and those with substantial missing case data.

**Figure 1:**
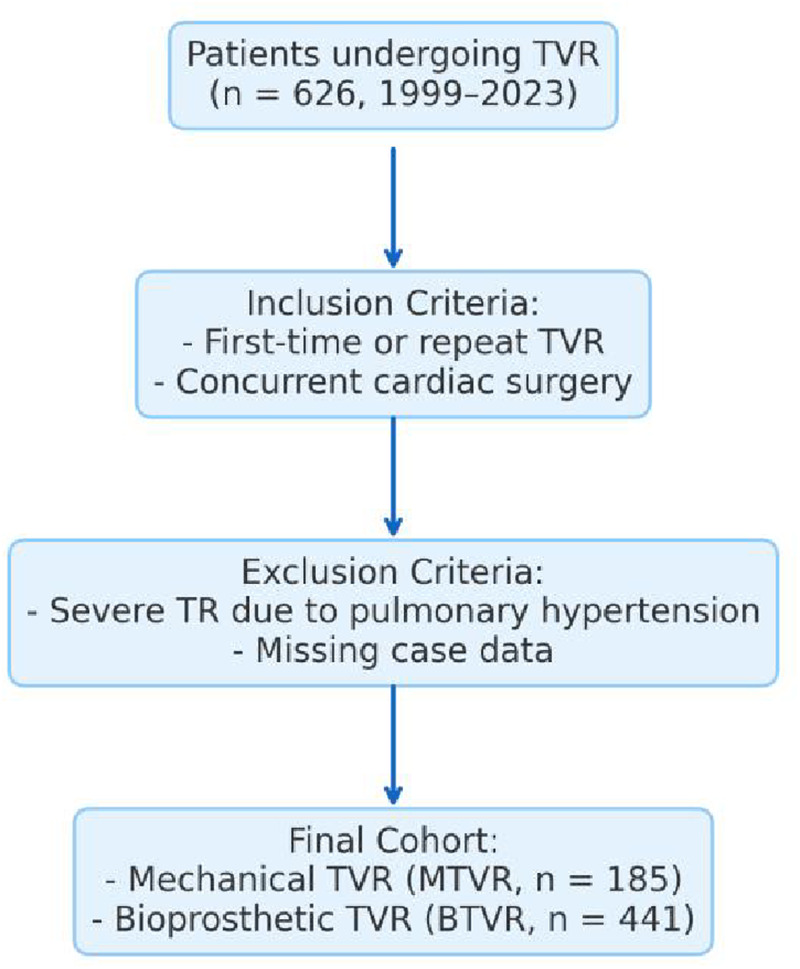
Flow diagram of patient enrollment.

A total of 626 patients underwent TVR from Jan.1st 1999 to Dec. 31th 2023. were enrolled. Among the enrolled patients, there were 207 males and 419 females, with an average age of 46.4 ± 13.5 years. Of these patients, 185 underwent mechanical tricuspid valve replacement (MTVR) and 441 received bioprosthetic tricuspid valve replacement (BTVR) (Figure 1).

### Endpoints

The primary endpoint was reoperation for tricuspid valve dysfunction or death from any cause. Secondary endpoints included early postoperative outcomes and long-term follow-up results. Early postoperative metrics included low cardiac output syndrome, IABP (Intra-Aortic Balloon Pump) implantation, ECMO (Extracorporeal Membrane Oxygenation) implantation, respiratory insufficiency, poorly healing wound, reoperation owing to bleeding, acute renal insufficiency, hemodialysis, compound hemorrhagic and thrombotic events, and in-hospital death. Long-term follow-up metrics included tricuspid valve condition, embolic events, bleeding events, permanent pacemaker implantation events, and cardiac function. Postoperative low cardiac output syndrome was defined as circulatory instability requiring a vasoactive inotropic score of >10 or the use of adjunctive circulatory support within 24 h of surgery. Compound hemorrhagic and thrombotic events were defined as active bleeding from any organ, subcutaneous bleeding, or symptoms of typical or atypical embolism. Respiratory insufficiency was defined as ventilator time exceeding 48 h. Poorly healing wound was defined as a wound that did not heal well within 14 days. Reoperation owing to bleeding was defined as reoperation because of active bleeding (drainage of fluid greater than 2 ml/kg/h for 2 h or more) after the operation. Acute renal insufficiency was defined as a blood creatinine value exceeding 133 µmol/L in the 24 h after the operation. Heart failure during follow-up was defined as hospitalization during which vasoactive drug support was required for symptoms of heart failure (chest tightness, shortness of breath, palpitations, bloating, and edema). Composite events I included low cardiac output syndrome, IABP implantation, ECMO implantation, respiratory insufficiency, poorly healing wound, reoperation owing to bleeding, acute renal insufficiency, hemodialysis, compound hemorrhagic and thrombotic events, and in-hospital death. Composite events II included embolic events, bleeding events, permanent pacemaker implantation events, Re-tricuspid surgery, Infectious endocarditis, heart failure and death during follow-up. In-hospital death was defined as mortality occurring within 30 days post-operatively or at any time during surgery. Follow-up death refers to mortality occurring 30 days post-operatively or after discharge if the hospital stay duration was 30 days or more. Valve regurgitation severity was graded on a scale of 0–4, with 0 indicating no regurgitation, 1 or 2 indicating mild regurgitation, 3 indicating moderate regurgitation, and 4 indicating severe regurgitation. Significant valvular regurgitation was defined as moderate or severe.

This study was approved by the Ethics Committee of the Guangdong Provincial People's Hospital (approval number: KY- Z - 2022–256–01). The requirement for informed consent was waived owing to the retrospective nature of the study.

### Surgical strategies

An echocardiographer independently assessed all pre- and post-operative echocardiography, collaborating with intraoperative findings to finalize treatment plan. Tricuspid valvuloplasty is usually the first option, while tricuspid valve replacement (TVR) is only considered for ongoing severe regurgitation or extensive valve damage.

Patients and their families were provided with clear information about the available valve options, including their benefits and drawbacks. Our center recommends bioprosthetic valves for patients aged >60 years and for young female patients with pregnancy needs.

The surgical approach typically involves a median thoracotomy, right-sided thoracic small incision, and percutaneous catheter. Additionally, concurrent procedures may involve aortic or mitral valve surgery, coronary artery bypass grafting, surgery for congenital heart disease, and cardiac tumor removal. Detailed pre-operative baseline data are presented in Table 1.

**Table 1:** Preoperative data of the study patients.

Variable	Overall *N* = 626	MTVR group [*N* = 185 (30%)]	BTVR group [*N* = 441 (70%)]	*P*-value
Age, y	48.00 [36.00, 57.00]	39.00 [30.00, 48.00]	52.00 [41.00, 59.00]	<0.001
Weight	53.00 [47.00, 60.00]	50.50 [45.00, 58.00]	54.00 [48.00, 61.00]	0.001
Female	419 (66.93%)	121 (65.41%)	298 (67.57%)	0.665
Atrial fibrillation	415 (66.29%)	102 (55.14%)	313 (70.98%)	<0.001
COPD	16 (2.56%)	0 (0.00%)	16 (3.63%)	0.005
Renal insufficiency^a^ (Preoperative)	6 (0.96%)	1 (0.54%)	5 (1.13%)	0.676
Hemodialysis	1 (0.16%)	0 (0.00%)	1 (0.23%)	>0.999
History of cardiac surgery	254 (40.58%)	57 (30.81%)	197 (44.67%)	0.002
Preoperative intensive care status	29 (4.63%)	1 (0.54%)	28 (6.35%)	<0.001
Hypertension	63 (10.06%)	14 (7.57%)	49 (11.11%)	0.231
Diabetes	19 (3.04%)	3 (1.62%)	16 (3.63%)	0.212
Poor mobility before surgery	46 (7.35%)	3 (1.62%)	43 (9.75%)	<0.001
NYHA class				0.009
I	8 (1.28%)	1 (0.54%)	7 (1.59%)	
II	191 (30.51%)	44 (23.78%)	147 (33.33%)	
III	291 (46.49%)	86 (46.49%)	205 (46.49%)	
IV	136 (21.73%)	54 (29.19%)	82 (18.59%)	
RA diameter (mm)		75.1 ± 17.7	82.2 ± 19.7	<0.001
RV (mm)		58.8 ± 10.2	59.6 ± 10.7	0.297
LVEF (%)		63.6 ± 9.0	62.7 ± 7.7	0.316
PASP (mm)		58.6 ± 22.6	50.7 ± 18.8	<0.001
TR (cm²)		20.1 ± 12.3	22.3 ± 11.4	0.028
CREA (µmol/L)		80.3 ± 21.4	80.4 ± 39.5	0.931
ALT (µmol/L)		39.6 ± 131.6	33.8 ± 97.8	0.515
Etiology of the tricuspid valve				<0.001
Rheumatic	385 (61.50%)	134 (72.43%)	251 (56.92%)	
Congenital	84 (13.42%)	6 (3.24%)	78 (17.69%)	
Infectious	97 (15.50%)	29 (15.68%)	68 (15.42%)	
Degeneration	49 (7.83%)	14 (7.57%)	35 (7.94%)	
Multiple	9 (1.44%)	1 (0.54%)	8 (1.81%)	
Ischemic	2 (0.32%)	1 (0.54%)	1 (0.23%)	
Tricuspid valve condition				<0.001
Regurgitation	443 (70.77%)	108 (58.38%)	335 (75.96%)	
Stenosis	3 (0.48%)	0 (0.00%)	3 (0.68%)	
Multiple	180 (28.75%)	77 (41.62%)	103 (23.36%)	

^a^ Renal insufficiency (Preoperative) was defined as blood creatinine value exceeding 133 µmol/L.

Values are presented as mean ± standard deviation [interquartile range] or *n* (%).

MTVR, mechanical tricuspid valve replacement; BTVR, bioprosthetic tricuspid valve replacement; COPD, chronic obstructive pulmonary disease; RA, right atrial; RV, right ventricular; CREA, creatinine; LVEF, left ventricular ejection fraction; ALT, alanine aminotransferase; PASP, pulmonary artery systolic pressure; NYHA, New York Heart Association; TR, tricuspid valve regurgitation.

### Follow-ups

Follow-ups were conducted through various means, including outpatient appointments, telephone calls, message communications, and assistance from public security authorities. Priority was given to utilizing the outpatient and inpatient medical record systems to gather essential information such as patient and family contact details, home address, and ID number. Patients were initially contacted via phone calls or messages to schedule follow-up visits at either their local clinic or our center for a comprehensive cardiac evaluation. Multiple attempts were made to maintain contact with the patients. Patients who could not be reached after multiple attempts were classified as “lost to follow-up.”

We retrieved and reviewed medical records, pre- and post-operative data, images, and operative notes from our local databases. Echocardiography was conducted by experienced specialists to assess ventricular function, size, and valve regurgitation, with a single echocardiologist reviewing all previous echocardiograms.

### Data analysis

Statistical analysis utilized SPSS version 27.0 (IBM Corporation, New York, NY, USA). Continuous variables are presented as mean ± standard deviation (SD), whereas categorical variables are presented as counts and percentages. The normal distribution of continuous variables was evaluated using the Shapiro–Wilk test. In the unmatched primary cohort, univariate analysis used the chi-square test for categorical variables and Mann–Whitney *U*-test for continuous variables. Survival analysis for the entire cohort was performed using Kaplan–Meier curves. Independent risk factors for in-hospital and overall death were evaluated using Cox logistic regression. A 1:1 nearest neighbor matching method for PSM was used. Nearest-neighbor matching was performed for variables such as age, gender, height, weight, atrial fibrillation, COPD Chronic Lung Disease, Cardiac Function, RA diameter, PASP and TR. Statistical significance was set at a two-sided *P*-value of <0.05.

## Results

### Evaluation of clinical outcomes

From 1999 to 2023, a total of 628 patients underwent TVR for tricuspid valve disease, but after identifying 2 duplicate cases, 626 patients were included in this retrospective study. After adjusting for propensity score matching (PSM), 142 matched pairs of cases were obtained. In total, 76 in-hospital deaths were recorded, with a mortality rate of 12.1%. The in-hospital mortality rate was significantly lower in the MTVR group than in the BTVR group (7.03% vs. 14.29%, *P* = 0.016), however, the difference disappears after performing propensity score matching (PSM).

The primary causes of tricuspid valve disease in the entire cohort were as follows: rheumatic changes, 392 cases (62.6%); congenital changes, 99 cases (15.8%); infections (including 18 drug-related), 88 cases (14.0%); degenerative changes, 36 cases (5.7%); ischemic changes, 7 cases (1.1%); and complex causes (including tumors, trauma, and pacemaker injuries), 4 cases (1.1%). A diverse range of valves was utilized in the surgeries. In the MTVR group, 35 Sorin Bicarbon Overline Valves, 1 CarboMedics Standard Valve, 4 On-X Mechanical Valves 25/33 mm, 3 Medtronic Standard Mechanical Valves (STD MV), 7 Medtronic ATS Mechanical Valves (MV), 6 domestic C-L short column tilting disc valves, and 129 St. Jude Medical Masters Mechanical Valves (MV) were used. In BTVR group, Carpentier-Edwards PERIMOUNT Valve was used in 125 cases, Carpentier-Edwards Magna in 15 cases, Medtronic Hancock II Valve in 268 cases, Medtronic Mosaic Bioprosthetic Valve in 28 cases, BVS Bioprosthetic Valve 4 cases, and JS-TTVI Device 1 case.

Significant differences in the etiological composition were observed between groups (*P* < 0.01). Furthermore, the BTVR group exhibited a higher proportion of patients with atrial fibrillation (*P* = 0.0002), an elevated New York heart function classification (*P* = 0.001), greater pre-operative mobility limitations (*P* = 0.0004), and more history of previous cardiac surgery (44.4% vs. 28.6%, *P* = 0.002) compared to the MTVR group. These findings suggest baseline data variation between groups. Detailed pre-operative baseline data were presented in Table 1.

In the MTVR group, The primary causes of death were low cardiac output syndrome and multiple organ failure in 8 cases, infectious shock (mainly pulmonary infection) in 1 case, cardiac arrest in two, and gastrointestinal hemorrhage in 2 cases. Conversely, the causes of death in the BTVR group included low cardiac output syndrome and multiorgan failure in 52 cases, infectious shock in 2 (endocarditis in 1 and lung infection in 1), cardiac arrest in 5, and gastrointestinal hemorrhage in 2. Additionally, one case of cerebral hemorrhage and cardiac rupture (intraoperative death) occurred, with no significant difference in the cause of death between groups (*P* = 0.114).

The overall incidence of post-operative acute renal insufficiency and hemodialysis was 9.9% and 11.7%, respectively. These rates were significantly lower in the MTVR group than in the BTVR group (4.3% vs. 12.2%, *P* = 0.004; 3.2% vs. 10.4%, *P* = 0.005). The MTVR group had more traditional median sternotomy approaches and the BTVR group had a higher proportion of minimally invasive approaches (*P* < 0.001) with more use of the beating-heart technique (*P* < 0.001). The proportion of isolated tricuspid valve surgeries was significantly higher in the BTVR group than in the MTVR group (*P* < 0.001). Furthermore, there were no significant differences in other post-operative parameters. These included the incidence of low cardiac output syndrome, the rate of secondary hemostasis, reintubation of tracheal tubes, cerebral complications, hemolysis, and the implantation of permanent pacemakers. After adjusting for PSM, hemodialysis rate was consistent with whole cohort result and the differences in the other variables disappeared. Detailed information is presented in Tables 2, 3.

**Table 2:** Operation data of the study patients.

Variable	MTVR group [*N* = 185 (30%)]	BTVR group [*N* = 441 (70%)]	*P*-value
Operative approach			<0.001
Median incision	178 (96.22%)	375 (85.03%)	
Endoscopic incision	7 (3.78%)	43 (9.75%)	
Other incisions	0 (0.00%)	23 (5.22%)	
Beating-heart technique	168 (90.81%)	349 (79.14%)	<0.001
Isolated tricuspid valve disease	48 (25.95%)	179 (40.59%)	<0.001

Values are presented as mean *n* (%).

**Table 3:** Operation outcomes of the study patients.

Variable	Variable before PSM				Variable after PSM			
	MTVR group [*N* = 185 (30%)]	BTVR group [*N* = 441 (70%)]	*P*-value	SMD	MTVR group [*N* = 142 (50%)]	BTVR group [*N* = 142 (50%)]	*P*-value	SMD
Low cardiac output syndrome	25 (13.51%)	83 (18.82%)	0.137	0.145	22 (15.49%)	21 (14.78%)	0.869	0.020
IABP	9 (4.86%)	23 (5.22%)	>0.999	0.016	7 (4.93%)	5 (3.52%)	0.555	0.070
ECMO	0 (0.00%)	5 (1.13%)	0.329	0.151	0 (0.00)	1 (0.70%)	1.000	0.119
Reoperation owing to bleeding	12 (6.49%)	40 (9.07%)	0.363	0.097	9 (6.33%)	16 (11.26%)	0.143	0.175
Reintubation	18 (9.73%)	26 (5.90%)	0.123	0.143	17 (11.97%)	6 (4.22%)	0.017	0.287
Respiratory insufficiency	22 (11.89%)	67 (15.19%)	0.340	0.097	19 (13.38%)	17 (11.97%)	0.721	0.042
Acute kidney insufficiency	8 (4.32%)	54 (12.24%)	0.004	0.290	8 (5.63%)	16 (11.26%)	0.088	0.204
Hemodialysis	6 (3.24%)	46 (10.43%)	0.005	0.288	6 (4.22%)	15 (10.56%)	0.041	0.244
Cerebral complication	1 (0.54%)	5 (1.13%)	0.676	0.065	1 (0.70%)	1 (0.70%)	1.000	0.000
Poorly healing wound	5 (2.70%)	16 (3.63%)	0.731	0.053	5 (3.52%)	9 (6.33%)	0.273	0.130
Other bleeding and embolic events	6 (3.24%)	9 (2.04%)	0.541	0.075	6 (4.22%)	2 (1.40%)	0.282	0.171
Pacemaker	4 (2.16%)	11 (2.49%)	>0.999	0.022	1 (0.70%)	4 (2.81%)	0.367	0.161
Infectious endocarditis (postoperative)	1 (0.54%)	1 (0.23%)	0.504	0.051	1 (0.70%)	0 (0.00)	1.000	0.119
In-hospital death	13 (7.03%)	63 (14.29%)	0.016	0.237	12 (8.45%)	18 (12.67%)	0.247	0.138
Composite event I	56 (30.2%)	155 (35.1%)	0.239	0.104	46 (32.39%)	51 (35.91%)	0.532	0.074

Acute kidney insufficiency was defined as blood creatinine value exceeding 133 µmol/L.

Values are presented as mean *n* (%).

IABP, intra-aortic balloon pump; ECMO, extracorporeal membrane oxygenation.

**Figure 2:**
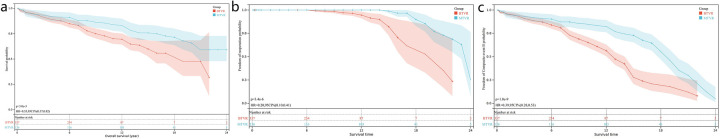
Kaplan–Meier curve and risk factor-adjusted curve based on Cox proportional hazards model for overall survival **(a)**, freedom of tricuspid reoperation **(b)**, freedom of Mace II **(c)** in the BTVR and MTVR groups. HR, hazard ratio; MTVR, mechanical tricuspid valve replacement; BTVR, bioprosthetic tricuspid valve replacement.

The study cohort included 514 cases with successful follow-up at Dec 15th to 31st 2023 and 33 cases lost to follow-up (23 from the mitral valve replacement group and 10 from the bioprosthetic valve replacement group), resulting in a follow-up rate of 93.9%, excluding in-hospital deaths. The median follow-up time was 11 years (range: 0.1–24 years), with a maximum follow-up time of 24 years. After excluding patients with missing values and those lost to follow-up, 550 patients were included in the survival analysis. The overall patient survival rates at 1, 5, 10, 15, and 20 years were 95.8% (95% CI: 94.0–97.6), 88.7% (95% CI: 85.7–91.6), 76.6% (95% CI: 72.7–80.7), 66.2% (95%CI: 61.3–71.5), and 56.6% (95%CI: 49.6–64.6), respectively. In the MTVR group, the survival rates at 1, 5, 10, 15, and 20 years were 95.5% (95% CI: 92.1–99.1), 91.8% (95% CI: 87.3–96.5), 85.6% (95% CI: 79.8–91.8), 75.1% (95% CI: 68.0–83.0), and 65.1% (95% CI: 56.0–75.6), respectively. In the BTVR group, the survival rates at 1, 5, 10, 15, and 20 years were 95.9% (95% CI: 93.8–98.0), 87.4% (95% CI: 83.9–91.0), 72.5% (95% CI: 67.5–77.8), 61.4% (95% CI: 54.4–69.2), and 32.5% (95% CI: 13.5–78.2), respectively. A significant difference was observed between groups in the survival curves of overall survival (HR 0.55, 95% CI: 0.37–0.82), freedom of tricuspid reoperation (HR 0.20, 95% CI: 0.10–0.41), freedom of Composite event II(HR 0.39, 95% CI: 0.28–0.53) (*P* *<* 0.01). Although no significant differences were observed in overall mortality and reoperation rates between the two groups in the matched cohort, survival curve analysis revealed a clear clinical advantage for the MTVR group. Based on the hazard ratio (HR = 0.55, 95% CI: 0.37–0.82) for overall survival, the MTVR group demonstrated significantly better survival compared to the BTVR group. Furthermore, the MTVR group exhibited lower risks for freedom from tricuspid reoperation (HR = 0.20, 95% CI: 0.10–0.41) and freedom from composite event II (HR = 0.39, 95% CI: 0.28–0.53). These results suggest that, despite no significant differences in overall mortality and reoperation rates, the MTVR group shows significant survival advantages across multiple key clinical outcomes, further confirming the superiority of its treatment efficacy (Figure 2).

During the follow-up period, 178 patients died [52 (32.1%) in the MTVR and 126 (29.2%) in the BTVR group]. However, no significant difference was observed in mortality between groups. The MTVR group had a higher rate of repeated tricuspid valve surgery and hemorrhage events during follow-up compared to the BTVR group (12.3% vs. 5.3%, *P* = 0.006; 6.9% vs. 5.1%, *P* = 0.008, respectively). Cardiac echocardiography results during follow-up showed that the MTVR group was more likely to experience valvular obstruction (10.8% vs. 2.7%, *P* < 0.01), whereas the BTVR group had a higher incidence of Intra-valvular regurgitation (19.4% vs. 43.3%, *P* < 0.01). The MTVR group had a significantly lower incidence of New York heart function classification III or IV (32.1% vs. 43.8%, *P* < 0.05), whereas the BTVR group had a significantly higher rate of heart failure admissions (4.32 vs. 14.15%, *P* < 0.01). In the matched cases, the differences of repeated tricuspid valve surgery, hemorrhage events and New York heart function classification III or IV were no longer present. Detailed information is presented in Table 4.

**Table 4:** Follow-up results.

Variable	Variable before PSM				Variable after PSM			
	Mechanical TV group [*N* = 162 (30%)]	Bio-prosthetic TV group [*N* = 431 (70%)]	*P*-value	SMD	Mechanical TV group [*N* = 142 (50%)]	Bio-prosthetic TV group [*N* = 142 (50%)]	*P*-value	SMD
Death during follow-up	52 (32.10%)	126 (29.23%)	0.564	0.029	39 (31.70%)	36 (25.71%)	0.283	0.133
Overall death	65/185 (40.1%)	189/441 (43.8%)	0.414	0.029	51 (41.46%)	55 (39.28%)	0.719	0.044
Thrombosis event	6 (3.70%)	12 (2.78%)	0.754	0.009	5 (4.06%)	2 (1.42%)	0.346	0.162
Re-tricuspid surgery	20 (12.35%)	23 (5.34%)	0.006	0.231	13 (10.56%)	8 (5.71%)	0.147	0.178
Hemorrhage event	41 (6.91%)	22 (5.10%)	0.008	0.180	15 (12.19%)	9 (6.42%)	0.105	0.199
NYHA class ≧III	52 (32.10%)	189 (43.85%)	0.012	0.280	39 (31.70%)	58 (41.42%)	0.103	0.203
Pacemaker	9 (5.56%)	27 (6.26%)	0.897	0.036	5 (4.06%)	12 (8.57%)	0.138	0.186
Infectious endocarditis	4 (2.47%)	8 (1.86%)	0.744	0.008	3 (2.43%)	3 (2.14%)	1.000	0.020
Heart failure admissions	7 (4.32%)	61 (14.15%)	<0.001	0.322	5 (3.57%)	20 (16.26%)	<0.001	0.434
Composite event II	95/185 (51.3%)	225/441 (51.0%)	0.106	0.076	66 (46.4%)	47 (33.1%)	0.02	0.233

Thrombosis and hemorrhagic events included any thrombosis or hemorrhage in any system of the body.

TV, tricuspid valve; NYHA, New York Heart Association.

Risk factor analysis revealed several perioperative indicators associated with patient mortality. To assess independent risk factors for in-hospital death, we used a Cox proportional risk regression model. In the model, postoperative in-hospital death was defined as an event variable and postoperative follow-up time as a duration variable. We controlled for potential confounders, including weight, New York heart function classification, extracorporeal circulation time, and ventilator time. Greater body weight significantly reduced the risk of early death compared with older age (HR = 0.94, 95% CI: 0.90–0.99, *P* = 0.17). Each one-grade increase in the New York heart function classification raised the risk of early death by 1.63 times (HR = 2.63, 95% CI: 1.39–4.97, *P* = 0.003). An increase of one minute in extracorporeal circulation time raised the risk of in-hospital death by 1%, while a similar increase in ventilator time raised it by 0.3% (HR = 1.01, 95% CI: 1.00–1.02, *P* = 0.009; HR = 1.003, 95% CI: 1.00–1.006, *P* = 0.038). During the follow-up period, New York heart function classification (HR = 3.45, 95% CI: 1.12–10.65, *P* = 0.031) was associated with long-term mortality. However the choice of valve was not an independent risk factor for perioperative or long-term mortality.

## Discussion

Tricuspid regurgitation is predominantly functional, with most cases attributable to rheumatic valves (62.6%) or infective endocarditis (14%) (6–8). Tissue damage and leaflet contracture from rheumatism or infection diminish the success rate of valve repair, leading to TVR in many instances (9).

The perioperative mortality rate of TVR surgery has been historically high (10–13), with our center reporting a peak rate of 23.5% (14, 15). With advances in minimally invasive technology and deeper insights into tricuspid valve disease, the perioperative mortality rate has gradually decreased to 12.1%, which is the same as that reported at home and abroad (9, 16, 17). The in-hospital mortality rate was significantly higher in the BTVR group than in the MTVR group, possibly related to the composition of both groups. Several factors contribute to this difference: the mean age of patients in the mechanical tricuspid valve replacement group was significantly lower than that in the bioprosthetic group. Additionally, there were notable differences in pre-operative underlying conditions, including atrial fibrillation, chronic obstructive pulmonary disease, and hypertension. Furthermore, the bioprosthetic group exhibited larger right atrial (RA) sizes and more severe tricuspid regurgitation compared to the mechanical group. Lastly, a larger proportion of patients in the bioprosthetic group had a history of previous cardiac surgery. Despite rigorous propensity matching, residual confounding from unmeasured variables (e.g., right ventricular function gradients or socioeconomic disparities in medication adherence) could persist. Second, historical biases on anticoagulation did exist: earlier-era patients receiving MTVR (1998–2008) may have faced less optimized anticoagulation regimens compared to later BTVR cases (2008–2023). Finally, the matched cohort's non-significant trend toward MTVR benefit suggests that true biological differences, if any, are likely larger than the initial estimate.

Our conclusion was that MTVR was significantly better than BTVR in terms of short and long-term survival. Some studies have suggested that the high mortality and complication rates of TVR compared with tricuspid valvuloplasty are not related to valve selection but rather to the patient's comorbidities, cardiac functional status, and nutritional status (18, 19). The obove points coincided with the results of our risk factor analysis. In this study, the New York heart function classification directly affected the short- and long-term prognosis of patients.

While our findings support MTVR as the preferred strategy for younger patients with long life expectancy, BTVR retains clinical relevance in specific populations: (1) Elderly patients (>70 years) with limited anticipated lifespan, where avoiding lifelong anticoagulation may reduce bleeding risks and improve quality of life; (2) Women of childbearing age desiring pregnancy, given the teratogenic risks of warfarin; and (3) Patients with contraindications to vitamin K antagonists. These considerations align with recent consensus statements advocating individualized valve selection (12). Concepts of valve selection has shifted from being based solely on age to incorporating various factors such as patient preference, acceptance of minimally invasive re-operation, quality of life, and physician recommendation (20, 21). In our earlier surgeries, patients were generally younger (average age <50 years), making mechanical valves the preferred option. However, over the past decade, bioprosthetic valves have become dominant. In our study, 70.4% of patients chose BTVR, aligning with common international practice (22). This shift could be attributed to several factors in our center. First, the advancement of medical technology in China has significantly reduced rheumatic valve lesions, while the population suffering from tricuspid valve disease is generally aging. Second, the promotion of minimally invasive laparoscopic technology has substantially reduced the surgical risk of BTVR. Although the difference was not significant, the in-hospital mortality rate in the minimally invasive endoscopic group was 4.2%, whereas that in the open group was 15.4%. Third, the robust Chinese economy has made BTVR the most popular option, as patients increasingly prioritizing their quality of life. Finally, the emergence of interventional valve technology has made bioprosthetic valves more suitable for valve-in-valve therapy than mechanical valves. Our center actively participates in domestic and international clinical trials on tricuspid valve interventions, including ongoing trials on domestic interventional valves. Although the results of MTVR were significantly worse than those of BTVR in terms of the rate of tricuspid valve reoperation, the rate of long-term bleeding events, and the incidence of heart failure, when combined with the time-series analyses, the survival curves, the cumulative freedom from tricuspid valve reoperation curves, and the cumulative freedom from Mace II curves of MTVR were significantly better than those of BTVR. This result was consistent with the results of a recent meta-analysis including 7,166 cases of tricuspid valve replacement by Sá MP et al. (22).

This study has some limitations. The single-center design, while avoiding confounding from cross-center practice differences, allowed us to establish granular data on valve-related outcomes over 24 years. This dataset may serve as a benchmark for future multicenter studies aiming to standardize surgical and postoperative management strategies. However during this period, there were five upgrades to the medical record system, reflecting the contributions of three generations of cardiac surgeons. This complexity introduces several difficulties. First the completeness and homogeneity of the case data cannot be guaranteed. Second, advancements in the valve manufacturing process, surgical techniques, and the differing perferences of surgeons may introduce unmeasured confounders. Surgeon-specific expertise and patient-level socioeconomic factors could also influence outcomes. Over the past decade, evolving surgical techniques over the 24-year study period (e.g.,transition from sternotomy to minimally invasive approaches) may have differentially impacted MTVR/BTVR cohorts. And long-term postoperative anticoagulation management has transitioned from a less rigorous approach to a strict requirement for monthly check-ins. This shift can also lead to some bias in anticoagulation-related outcomes. However, we attempted to present authentic data to demonstrate the long-term survival of patients who underwent TVR in the our center. Despite the imperfections in our results and statistical analyses, we endeavored to improve the data through several rounds of follow-up to present the real long-term survival of patients who underwent TVR in South China.

## Conclusions

A mechanical valve in the tricuspid position may offer advantages over a bioprosthetic valve in terms of short- and long-term survival. Body weight, New York heart function classification, extracorporeal circulation time, and ventilator time were identified as independent risk factors for in-hospital mortality. New York heart function classification during follow-up was identified as an independent risk factor for overall patient mortality.

## Data Availability

The original contributions presented in the study are included in the article/Supplementary Material, further inquiries can be directed to the corresponding authors.
